# IdentIRCCS. The description of healthcare professionals employed within IRCCS University Hospital of Bologna

**DOI:** 10.3389/fpubh.2025.1599845

**Published:** 2025-09-16

**Authors:** Martina Giusti, Silvio Quirini, Angela Peghetti, Stefano Durante

**Affiliations:** ^1^Department of Experimental and Clinical Medicine, University of Florence, Florence, Italy; ^2^Steering Committe, Centro Studi SAPIS Foundation, Italian National Federation of Orders of Radiographers and Technical, Rehabilitation, and Prevention Health Professions Research Centre, Rome, Italy; ^3^SPIR, IRCCS Azienda Ospedaliero-Universitaria di Bologna, Bologna, Italy

**Keywords:** welfare, management, health professionals, decision-making, data analysis

## Abstract

Globally research centres in healthcare sector express the excellence in the translational medicine, from the evidence-based medicine to the evidence-based practice. In Italy, Institutes of Hospitalization and Healthcare with Scientific Goal (called IRCCS) are recognized as the driving force of innovation within healthcare sector. It is reasonable to expect that health professionals working within IRCCS institutions should embody professional excellence in a setting characterized by technological and organizational excellence. Nevertheless, in Italian public IRCCS, the personnel are selected by public competitions without any specific requirements in relation with the specific working context. According to the current state-of-art, this research aims to investigate the characteristic of healthcare professionals currently employed within Italian IRCCS facilities. IRCCS University Hospital of Bologna was chosen as suitable case study due to the enlargement of top management within the Healthcare Professions Directorate, that manifests a particular sensibility toward healthcare professionals growing. Dataset on personnel included sociodemographic information (age, sex, place of birth, residence, domicile), professional details (job category, role, working hours, training, allowances, contribution), and training status (household composition, number of children, number of dependents, physical limitations). The detailed description of healthcare professionals employed within the case study supports evidence-based decision-making for the development of corporate welfare policies and of targeted management strategies, useful and applicable in each healthcare research centre worldwide.

## 1 Introduction

Globally research centres in healthcare sector are the promoters of scientific progress by a theoretical perspective. These centres play a pivotal role in advancing medical knowledge, improving patient outcomes, and fostering innovation in health systems. At the heart of these institutions lies the critical contribution of researchers, who serve as the linchpins of research translation into clinical practice (1, 2).

On the other hand, university hospitals have the responsibility of translational medicine, transitioning the new discoveries in health care from the evidence-based medicine to evidence-based practice (3, 4). A persistent challenge is the limited interaction and collaboration between healthcare professionals working in research centres and their colleagues employed in healthcare organizations (2, 5, 6). Possible solutions to this challenge have been the establishment of collaborative models, such as the involvement of researchers embedded in practice to align research studies with the specific concrete knowledge gaps or the engagement of health professionals in research (7) to ensure the acquisition of relevant and sustainable knowledge applicable in practice (1, 2, 8). Moreover, the integration of health professionals into research teams fosters the co-creation of knowledge, bridging the traditional divide between theory and practice among medical researchers and clinicians (9). Selection and recruitment strategies into health care organizations to enrol healthcare professionals interested in research can be a key point to favour this interaction (10).

Into the health workforce, healthcare professionals have often faced barriers to active participation in research, including limited research training, and institutional challenges despite their critical role (11). Overcoming these barriers have globally required targeted efforts to build research capacity among health professionals, providing them with the skills (i.e., translational researchers prefer short, easily accessible, and interactive training sessions during the working day) (12), resources (access to database, statisticians and data analysist, money for publication, etc.) (10), and operative support (health facilities oriented to research and organized and managed to achieve this goal) (13, 14) necessary to engage in high-quality research endeavours.

In the Italian context too, there are increasingly more healthcare professionals who possess the necessary skills to envision the world of research as a career opportunity having obtained a Ph.D., on par with their European, Anglo-Saxon, or American colleagues (15–17). Moreover, the growing presence of Institutes for Hospitalization and Healthcare with a Scientific Objective (IRCCS—with public or private nature) expands the opportunities for these professionals to combine their professional activity with their desire to engage in research. In fact, IRCCS have the institutional mission to provide treatment activities of excellence and to research on them (18). To achieve these purposes, IRCCS have a specific research management, integrating the own health workforce with the “Pyramid research” according with Law 205/2017 (19) and Ministerial Decree 164/2019 (20). The pyramid research is composed of health research staff and professional health research collaborators dedicated to research, equal to 35% of the entire staff working in the IRCCS, selected by specific calls in the research field and not involved in health care in accordance with international standards.

The coexistence of the “Pyramid research” within the health workforce of IRCCS raises two critical issues.

The first issue is the ongoing division between research and professional activities among health professionals in IRCCS The second issue is that 65% of health professionals employed in IRCCS cannot engage in research and advanced specialization because their recruitment and selection through regional public competitions do not allow for the inclusion of specific requirements related to their specific working context (21, 22).

It seems to revert to the starting point; however, it is not demonstrated by in literature reviews.

In fact, there is a knowledge gap in investigating the characteristics of health workforce employed in public research centres and university hospital, like the Italian IRCCS. For this reason, this research aims to investigate the characteristic of all healthcare professionals currently employed within Italian IRCCS facilities to develop more effective approaches for human resources management. Identifying socio-demographic characteristics, professional details and education and training status of the healthcare professionals employed in IRCCS should enable evidence-based decision-making for the development of corporate welfare policies and of targeted human resources management strategies, focused on competences and skills rather than tasks, useful and applicable in each healthcare research centre worldwide.

## 2 Material and methodology

The case study was identified as the most suitable methodology for pursuing the goal of this research (23, 24). This methodology facilitated the in-depth understanding of a phenomenon within intervention context (25), especially when the object of analysis is complex as the study and the analysis of integrated management of economic and professional resources in the assistance of frail patients. Among several IRCCS presented in Italy, the public IRCCS University hospital in Bologna was chosen as significant case study for many reasons. The first is that this IRCCS was established few years as IRCCS so only nowadays it is fully up to speed. The second reason is the nature of the University Hospital of Bologna, one of the main publics, non-specialized polyclinics in Italy, which is recognized for its excellence in healthcare through the establishment of an IRCCS in a specific thematic area. The third is the presence of Health Professions Directorate deputed to concur to the definition and the reaching of strategic mission of health organization, to guarantee the overall governance of the assistance, rehabilitation, technical-health and prevention functions. Besides, a key feature of this methodology is also its ability to examine phenomena from a practical perspective, addressing one of the most discussed issues in corporate literature: the gap between theory and practice (26). Ultimately, the case study approach facilitates the generalization of findings, broadening their applicability to comparable scenarios (27).

However, while case studies can offer in-depth insights into complex organizational phenomena, their generalizability is often debated. As noted by Crowe et al. (28), “case study research has sometimes been criticised for lacking scientific rigour and providing little basis for generalisation.” Similarly, Yin (29) highlights that generalization from case studies must be approached with caution. Therefore, the findings from this case study should be interpreted as context-specific, while potentially offering transferable insights to similar healthcare settings.

In fact, the investigation of the IRCCS University Hospital in Bologna may offer useful insights for informing workforce strategies in other health research centres and university hospitals with similar organizational and institutional characteristics.

### 2.1 Data collection and analysis

Given the exploratory and descriptive design of the study, the analysis did not focus on a single dependent variable. However, key outcome variables—such as employment status, contract type, working time, and educational level—were examined in relation to socio-demographic and professional characteristics (age, sex, length of service, job role). No formal confounding control was applied, and findings are to be interpreted as observational and descriptive.

Data were collected through a formal request to the Director of Health Professions Directorate of IRCCS University Hospital in Bologna.

About data regarding the employment status of all non-executive healthcare professionals employed at the IRCCS University Hospital in Bologna, the director provided to the research group database extracted by the regional personnel administration system (called GRU^1^). The database contained pseudo-anonymized data regarding the employment status of all non-executive healthcare professionals employed at the IRCCS University Hospital in Bologna at 12/31/2023.

It was not required the approval of an institutional bioethical committee due to the organizational nature of the investigation and the direct responsibility assumed by Director on the data sharing. Moreover, received aggregated data do not allow to identify nobody belonging to IRCCS University Hospital of Bologna.

About data on educational and training status of health professionals employed at the IRCCS University Hospital of Bologna, the director provided the research group with the final results of a company questionnaire. This questionnaire was administered to all staff members during their annual evaluation in mid-2024, using a census approach rather than random sampling. All employees were invited to participate. However, no follow-up analysis was performed to assess potential response bias among non-respondents. Therefore, the results reflect the perspectives of respondents only and may not fully represent the entire workforce. The survey was designed to collect detailed information regarding academic qualifications, post-graduate education, and additional certifications.

Data were analysed using descriptive statistics (frequencies and percentages) for categorical and continuous variables. Due to the descriptive purpose of the study and limitations in data structure, no multivariate analysis was performed.

No inferential statistical tests (e.g., chi-square, *t*-test, or regression models) were performed, as the study's primary aim was to provide a comprehensive descriptive overview of the healthcare workforce rather than test specific hypotheses. As such, comparisons between groups (e.g., gender and professional category) are presented narratively and visually, without formal significance testing. This limits the analytical depth of the study and prevents drawing conclusions about significant differences between subgroups.

Results were discussed in accordance with evidence from literature review.

## 3 Results

### 3.1 IRCCS University Hospital of Bologna

The object of study has been to analyse the IRCCS University Hospital of Bologna, recognized by the Italian government decree dated September 19, 2020, in the thematic areas of assistance and research in transplants and critical patients and integrated medical and surgical management of oncological pathologies.

The designation as an IRCCS in 2020 marked the apex of this evolution, confirming its role as a leading institution in scientific research and medical specialization. In the field of research, the IRCCS University Hospital of Bologna excels with projects ranging from transplants to the management of oncological and critical pathologies. With an investment of over €34,472,109 in active research projects in 2023 and additional ministerial and private funding supporting advanced research, the hospital continues to be at the forefront of developing new therapies and technologies. The scientific output in 2023 includes 1,969 publications in specialized journals with a total Impact Factor of 11,052.1, demonstrating the impact and importance of the research conducted within the institution.

Today, the IRCCS University Hospital of Bologna boasts 1,515 beds and employs a staff of 6,807, including 1,076 researchers and 212 professional collaborators dedicated to research. Annually, it records approximately 49,000 hospitalizations and over 3,300,000 specialist services. The hospital is structured into several integrated pavilions and university departments, with 87 specialized operational units offering cutting-edge treatments across a broad range of medical and surgical disciplines.

The relationship with the University of *Bologna* is one of the distinctive aspects of IRCCS University Hospital of Bologna. Additionally, this institution has agreement with 123 universities in Italy and collaborates with numerous international universities, providing a training ground for future healthcare professionals, including doctors, nurses, midwifes and, allied health professionals. This collaboration fosters intense research and teaching activities, with daily participation of university students and professors who are actively involved in clinical practice and scientific studies.

### 3.2 Data on healthcare professionals employed within IRCCS University Hospital of Bologna

On December 31st, 2023, the non-managerial healthcare personnel in service at the IRCCS University Hospital of Bologna amounted to 3,938 healthcare professionals.

The data analysis focused on 3,567 active employees under the Health Professions Directorate.

A total of 210 professionals (5.3%) were excluded from the data analysis as they belonged to other corporate Directorates: General Directorate (9), Administrative Directorate (63), Health Directorate (113), General Cost Structures (20), and Scientific Directorate (5).

Seventy-three percentage (2,617) of the professionals recognized themselves as female, while the remaining 27% (950) as male. No professional has requested the registration of another gender identity into GRU.

The average age of the sample was 45.9 years. 10.8% (387) were between 20 and 29 years old, 28.4% (1,022) were between 30 and 39 years old, 24.4% (873) were between 40 and 49 years old, 28.3% (1,007) were between 50 and 59 years old, and the remaining 8.1% (289) were between 60 and 70 years old.

In 2024, 161 professionals retired (Table 1).

**Table 1:** Sex and age of healthcare personnel in IRCCS University Hospital of Bologna.

**Age**	**Females**	**%**	**Males**	**%**
21–30 years	359	13.7	82	8.6
31–40 years	696	26.8	286	30.2
41–50 years	603	23.0	280	29.5
51–60 years	774	29.5	230	24.2
61–66 years	185	7.0	72	7.5
Total	2.617	73.4	950	26.6

Although formal inferential statistical tests were not conducted, a visual inspection of the data suggests age distributions differ slightly by sex, with a higher proportion of males in older age brackets (41–60 years). This may reflect historical sex imbalances in certain healthcare roles that have gradually shifted in recent decades.

Ninety-nine percentage of the sample, equivalent to 3,528 individuals, held Italian citizenship. The second most represented citizenship in the sample was Romanian (12), followed by Polish (6).

From a geographical perspective, the province of Bologna represented the residence of most of the personnel, with 2,932 professionals residing there (82.1%). However, a significant figure was represented by 502 professionals (14%) residing outside the Emilia-Romagna region, of whom 219 had less than 2 two years of length of service.

The professionals had an average length of service of 12.7 years. 43.2% (1,542) of the analysed sample had a length of service of 0–5 years, 9.1% (324) of 6–10 years, 7.2% (257) of 11–15 years, 11.6% (414) of 16–20 years, 10.7% (382) of 21–25 years, and 18.2% (648) of 26–30 years. Focusing on the first bracket, only 87 professionals had a length of service of less than 1 year, with an average age of 30 years (Table 2).

**Table 2:** Age and length of services of healthcare personnel in IRCCS University Hospital of Bologna.

**Age**	**Length of service (years)**						
	<**1**	**1–5**	**6–10**	**11–15**	**16–20**	**21–25**	**26–30**
21–30 years	59	442	7	//	//	//	//
31–40 years	17	659	165	99	20	//	//
41–50 years	10	225	104	106	277	135	27
51–60 years	1	114	38	40	98	217	494
61–66 years	//	15	10	12	19	30	127
Average age	30	36	41 years	45	47.5	51	57

97.5% of the sample (3,477) had a full-time work. Of the remaining 2.5% (90), they had vertical, horizontal, and cyclical part-time work. 99.2% (3,529) had an open-ended employment contract.

There were only 21 integrated university graduates, specifically 11 biomedical laboratory technicians, five radiographers, two midwives, one nurse, and one healthcare assistant. There were only five professionals with a fixed-term employment contract (Table 3).

**Table 3:** Professional details of healthcare personnel in IRCCS University Hospital of Bologna.

**Profession**	**No**.	**Sex**		**Av. age**	**Av. length of service**	**Working time**	
		**Females**	**Males**			**Full-time**	**Part-time**
Nurses	2,234	1,667	567	43.2	13.9	2,187	47
Radiographers	158	82	76	44.1	13.9	151	7
Technician of biomedical laboratory	125	97	28	43.7	13.5	120	5
Midwifes	62	62	//	38.7	10.8	62	//
Physiotherapists	52	39	13	47.1	14.1	50	2
Other professionals	84	69	15	42.7	13.1	78	6
Health care assistant	852	601	251	47.9	10.9	829	23

There appears to be a concentration of part-time contracts in specific professional roles (e.g., nurses and healthcare assistants), potentially reflecting caregiving responsibilities, which may disproportionately affect female staff. This trend merits further investigation in light of gender-sensitive human resources policies.

Moreover, the overwhelming predominance of permanent, full-time contracts across all professional categories suggests high institutional stability. However, this configuration may reduce organizational flexibility, especially in the face of evolving clinical and research demands.

A total of 2.575 individuals completed the questionnaire assessing the educational and training status, out of 3.938 employees, representing 65.4% of the total workforce (Table 4).

**Table 4:** Educational and training status of healthcare professionals.

**Profession**	**No**.	**Equivalence**	**Bachelor degree**	**Master degree**	**Professional course I/II level**	**Improvement courses**	**PhD**	**Other**
Nurses	1,537	539	998	141	501/14	162	7	41
Radiographers	146	47	99	39	78/5	7	//	10
Technician of biomedical laboratory	119	22	97	28	22/1	6	4	4
Physiotherapists	39	18	21	8	16/0	0	//	3
Midwifes	73	9	64	10	33/1	15	//	2
Other healthcare professionals	84	33	51	23	26/2	12	//	8

Among the respondents, 1.998 were healthcare professionals and 577 were health care assistant.

While the majority of professionals hold bachelor's degrees or equivalent, the relatively low number of master's and PhD holders may limit the institution's capacity to fully integrate research into clinical practice. This is particularly relevant for IRCCS settings, where research and innovation are central to the mission.

A special mention must be done for the figure of health care assistant, who was established with the agreement of 22/02/2001 (“Agreement between the Ministry of Health, the Ministry for Social Solidarity, the Regions and the Autonomous Provinces of Trento and Bolzano”). This health profile carries out its activity independently in basic patient care, respecting the tasks dictated by its professional profile. Its formation is not academic, but responsibility of the Regions and Autonomous Provinces so is not recognized formally as healthcare professional in Italy.

The survey response rate was 68.8% among nurses, 92.4% among radiographers, and 95.2% among laboratory technicians. Midwives achieved a 100% response rate, while 75% of physiotherapists completed the survey. A total of 66.6% of professionals hold a bachelor's degree, while approximately one-third (33.4%) possesses qualifications recognized as equivalent to the current bachelor's degree, such as pre-reform diplomas or foreign-recognized titles.

The analysis of the educational pathways also highlighted those 699 professionals, representing 27.1% of all respondents, had completed a professional course (both of I and II Level) aimed at enhancing their technical and specialized competencies. Additionally, 202 professionals (7.8%) have attended improvement courses, designed as short-term training initiatives for professional development.

The percentage of professionals who have obtained a master's degree remains limited (9.6%), and only 0.4% (11) hold a PhD.

A total of 24 nurses obtained their professional qualification abroad (one in Spain, three in South America, and 20 in Asia), and 137 professionals hold additional degrees not related to the healthcare field. These include, for example, certifications such as National Chess Instructor, Conservatory Diploma, and university degrees in Fine Arts, Physics, Economics, Natural Sciences, Archaeology, and other disciplines.

## 4 Discussion

The discussion has been structured to reflect the key findings presented in the results section, highlighting sex distribution, workforce aging, contract types, and educational qualifications. Each theme is interpreted in light of relevant literature and compared with trends observed in other health systems, where applicable.

The analysis of the healthcare workforce at the IRCCS University Hospital of Bologna offers meaningful insights into the organizational and professional profiles of staff within a high-specialization, research-oriented hospital. The current workforce configuration, recruitment methods, and the integration of research into clinical practice highlight systemic challenges and opportunities for improvement.

### 4.1 Socio-demographic data

The significant female predominance (73%) among healthcare professionals aligns with global trends, where the feminization of the healthcare workforce is a well-documented phenomenon (30). However, this highlights the need to rethink corporate welfare policies, as female healthcare professionals often face unique challenges in balancing work and family life. These challenges can impact career progression, job satisfaction, and consequently, workforce stability (31). Another key finding is the high average age of the workforce (45.9 years), with 36.4% of employees over 50 years old. This raises concerns about the long-term sustainability of the system, given the potential impact of retirements and the need for a structured generational turnover (32). Internationally, strategies to address this challenge include incentives for senior professionals to remain in service and facilitated pathways for young healthcare workers (33). In Italy, however, workforce aging policies in healthcare remain fragmented (34).

A critical aspect related to the aging workforce is the potential decline in physical performance. As healthcare professionals grow older, they may experience reduced physical endurance, slower recovery from fatigue, and a decline in their ability to handle physically demanding tasks, such as lifting patients, prolonged standing, or responding to emergencies in high-stress environments like intensive care units. These challenges can impact both individual performance and overall healthcare efficiency, particularly in hospital settings that require constant physical engagement (35). To mitigate these risks, ergonomic workplace adjustments, task redistribution strategies, and targeted wellness programs have been adopted in various international healthcare systems (36).

Recently, the IRCCS University Hospital of Bologna has become one of the first institutions in Italy to implement an ergonomics program, aimed at supporting aging professionals in maintaining their productivity while reducing physical strain. This initiative includes ergonomic training, workplace modifications, and assistive technologies designed to optimize movement efficiency and minimize work-related musculoskeletal disorders. The implementation of this program represents a significant step toward aligning Italian healthcare institutions with international best practices, demonstrating a growing awareness of the importance of workforce wellbeing in sustaining high-quality patient care (37). While this initiative aligns with international best practices, its impact has not yet been formally evaluated in peer-reviewed studies. Preliminary institutional reports suggest positive staff feedback, but further research is needed to assess measurable outcomes.

Moreover, the relatively high average age of the workforce is also a consequence of employment shifts caused by the COVID-19 pandemic. During the pandemic, many healthcare professionals resigned from private healthcare facilities to seek employment in the public healthcare system, primarily due to greater job security, better benefits, and improved working conditions compared to the private sector (38). This exodus from private to public institutions has contributed to an aging workforce in public hospitals, as older professionals with significant experience sought more stable career opportunities during a time of unprecedented crisis (39). While this shift has strengthened the expertise available in public healthcare, it also contributed to an aging workforce, highlighting the urgent need for structured renewal and retention strategies.

A distinctive feature of the Italian scenario is that 14% of healthcare professionals employed at the IRCCS University Hospital of Bologna reside outside the Emilia-Romagna region. This could indicate the hospital's attractiveness as a centre of excellence. However, it also highlights that a considerable proportion of the workforce resides outside the Emilia-Romagna region. This fact could suggest that professionals living outside the region might be more inclined to leave their positions in favour of jobs closer to home or more convenient opportunities elsewhere. This geographical dispersion may increase turnover risk, especially for hard-to-replace professionals with specialized skills. Addressing retention in this subgroup is crucial to ensure continuity of care (40).

### 4.2 Professional details

The high proportion of professionals with more than 16 years of service (40.6%) suggests strong job stability. While this can be seen as a positive factor—ensuring continuity of care and the consolidation of expertise—it may also present challenges if stability translates into reduced flexibility in adapting to new organizational and technological models (32). Globally, advanced healthcare systems have addressed the challenge of workforce stability by balancing experience with innovation. In the UK's National Health Service, for example, upskilling programs and transition schemes toward academic or leadership roles for senior staff have been implemented to encourage greater internal mobility (41). In contrast, in Italy, continuing education is often left to individual initiative, with limited institutional incentives to ensure continuous professional development (42). The high rate of permanent contracts (99.2%) and full-time employment (97.5%) reinforces the picture of workforce stability. This echoes findings in European public healthcare systems where contractual stability is often used to retain skilled staff, particularly in high-pressure settings such as teaching hospitals. However, without effective work-life balance policies, this configuration may lead to increased burnout and reduced organizational flexibility (43).

At the same time, it is crucial to develop recognition policies that highlight the value of senior professionals. The analysis revealed that a significant portion of the staff is aged between 50 and 59 years (28.3%), and many professionals have accumulated extensive experience with an average length of service of 12.7 years. These senior professionals represent a key resource for the institution due to their expertise and experience. However, their motivation and retention in the system also depend on how their roles are recognized.

Recognition strategies, such as mentoring or knowledge transfer programs, could support the motivation and retention of experienced professionals.

In summary, to address the challenges revealed by the data analysis, it is essential to adopt an integrated approach that promotes continuous professional development, increases involvement in research, and adequately recognizes the contribution of senior professionals. Such initiatives will not only improve work quality and staff satisfaction but also strengthen the IRCCS University Hospital of Bologna as a centre of excellence in healthcare training, research and innovation.

#### 4.2.1 Focus on health professionals' composition

The composition of the healthcare workforce at the IRCCS University Hospital of Bologna reflects a model widely adopted across the Italian healthcare system. Nurses constitute the majority (62%) of healthcare professionals, followed by healthcare assistants (21.6%) and technical-health professionals such as radiology and laboratory technicians. While this distribution ensures a clear delineation of roles within clinical practice, it also differs substantially from the organizational structures found in other European and North American healthcare systems (44).

In Scandinavian countries and the UK, for example, nurses are granted a more advanced scope of practice, allowing them to take on tasks that in Italy are often delegated to health care assistant. These advanced nursing roles, such as nurse practitioners (NPs) and advanced practice nurses (APNs), are increasingly responsible for patient assessment, medication administration, and even minor medical procedures, reducing the reliance on support roles (34).

One of the most distinctive aspects of the Italian healthcare system is the presence of health care assistant (45). A critical issue is that the health care assistant's role lacks a direct equivalent in many other healthcare systems, where nurses themselves often perform similar functions (46). For this reason, there is not strong evidence on their contribution in assistance. In Anglo-Saxon countries, for example, health care assistants and nursing aides undertake comparable duties but follow structured training pathways that allow for clear career progression into registered nursing roles (47). In France and Germany, support roles similar to health care assistant exist, but their training programs are more structured, and they have greater opportunities for professional advancement (48).

### 4.3 Education and training status

The educational background of healthcare personnel reflects the high average age of the workforce, with many professionals qualified under pre-reform systems. This demographic and educational profile highlights the need for targeted educational and professional development strategies aimed at updating and aligning competencies with the current clinical, organizational, and research standards required in highly specialized healthcare settings.

In particular, the data regarding the proportion of professionals with advanced qualifications, such as master's degrees and PhDs, is relatively low, with only 9.6% of professionals holding a master's degree and an even smaller percentage (0.4%) holding a PhD.

These figures highlight a structural mismatch between the advanced academic profile required to contribute actively to IRCCS research missions and the current educational background of most staff. Moreover, the fact that participation in advanced training programs appears uneven across professional groups suggests an opportunity to tailor development initiatives based on role-specific gaps and institutional priorities.

This underscores a significant gap in advanced academic preparation, which is crucial to align professional competencies with the institution's mission of research and innovation (40). Given that the IRCCS University Hospital of Bologna is strongly research-oriented, it is essential to increase the proportion of staff with advanced academic qualifications. This would not only ensure alignment with the competencies required in an innovative environment but also promote the integration of scientific evidence into clinical practice—an essential aspect of improving care quality and advancing medicine.

Examples of successful strategies from the UK show how advanced qualifications can be integrated into healthcare systems effectively (34).

Furthermore, the fact that only one professional is currently officially employed in the pyramid research highlights a significant gap between clinical roles and research involvement. This disparity suggests the opportunity to review career pathways, creating new opportunities that allow staff to integrate clinical practice with research activities. This would not only contribute to the hospital's research mission but also provide professional stimulation and growth opportunities, encouraging greater participation in generating new scientific knowledge.

Furthermore, the analysis indicates that a large number of professionals are participating in various continuing education courses. While these programs contribute to professional development, it is essential to note that many of the courses offered do not align directly with the specific clinical and organizational needs of the institution. This disconnect suggests to tailor educational offerings to better match the needs of the workforce. Continuing education programs should be closely aligned with the current and future requirements of the IRCCS, ensuring that training not only enhances individual competencies but also supports the organization's strategic goals. A strategic alignment between educational programs and organizational objectives is crucial for maximizing the impact of training investments. This alignment will foster improvements in clinical outcomes, enhance staff satisfaction, and contribute to the overall quality of care. By addressing these gaps in training and ensuring that professional development is closely tied to the organization's mission of research, innovation, and patient care, the IRCCS can further strengthen its workforce, improve stability, and enhance the quality of services provided.

### 4.4 General considerations in relation to the IRCCS setting

Regarding the characteristics of healthcare personnel working in an IRCCS, it is essential to describe the modalities of personnel engagement, as these play a crucial role in ensuring effective integration between clinical practice and scientific research. Indeed, the level of personnel engagement directly influences the institution's ability to foster innovation, improve healthcare outcomes, and maintain high standards of scientific research. Effective engagement strategies ensure that healthcare professionals are actively involved in both patient care and research activities, facilitating a seamless translation of scientific advancements into clinical practice. Moreover, structured engagement fosters professional development, enhances job satisfaction, and contributes to workforce stability, which is particularly crucial in research-intensive healthcare settings such as IRCCS institutions. One of the key structural limitations of the Italian healthcare system lies in its uniform recruitment process, which applies identically to all public healthcare institutions, including IRCCS centres. Currently, healthcare professionals in IRCCS institutions are hired through regional public competitions, which assess candidates based on generalized qualifications and standardized examination procedures (49).

While this approach ensures transparency and meritocracy, it does not account for the specific mission of IRCCS institutions, which combine clinical care with translational research. In other advanced healthcare systems, research hospitals and academic medical centres employ more flexible, research-oriented hiring strategies that prioritize candidates with strong backgrounds in clinical research, academic achievements, and specialized training (40).

For example, in the United Kingdom, leading Academic Health Science Centres (AHSCs) such as Imperial College Healthcare NHS Trust and King's Health Partners incorporate clinical research experience, academic qualifications (e.g., Master's and PhDs), and demonstrated contributions to scientific publications as key selection criteria in their hiring processes. Similarly, in the United States, institutions like the National Institutes of Health (NIH) Clinical Center prioritize recruitment based on research competencies and methodological skills, ensuring that staff are well-equipped to contribute to both patient care and scientific advancements (50–52).

Although Law 205/2017 (19) and Ministerial Decree 164/2019 (20) introduced provisions for IRCCS centres to allocate up to 35% of their workforce to research-exclusive contracts, this reform has not been fully implemented. Most IRCCS professionals continue to be hired under the standard Italian health system recruitment framework (49). This misalignment between hiring policies and institutional objectives limits the ability of IRCCS institutions to attract and retain professionals with strong research backgrounds, ultimately hindering the integration of scientific innovation into clinical practice. Addressing this gap may require reconsidering recruitment frameworks, with attention to research experience, academic qualifications (such as master's degrees and PhDs), and alignment with institutional objectives. Of course, these considerations are extremely shaped, and maybe limited also, by the normative and organizational context that characterises the Italian healthcare systems and the role of healthcare professional within it.

However, such measures would promote IRCCS institutions to function within the Italian healthcare system as true centres of excellence, where clinical expertise and research innovation coexist seamlessly, in accordance with the international best practices (53).

## 5 Conclusions

This study highlights that healthcare professionals currently employed in the Italian IRCCS are typically female, with an average age of over 40, working full-time with permanent contracts, and holding a bachelor's degree. According with these findings, personnel management policies aimed at identifying and implementing corporate welfare solutions tailored to this specific target group are essential. These policies should focus on addressing the specific needs of healthcare professionals employed in IRCCS, ensuring they receive appropriate support in terms of work-life balance, career development, and overall wellbeing. By offering tailored welfare benefits, organizations can enhance not only employee satisfaction, reduce turnover, and promote a more sustainable and supportive work environment but also productivity, performance, and sustainability. Moreover, the findings reveal disparities in the educational and career opportunities available to staff.

While the growing number of healthcare professionals with advanced qualifications and a passion for research is promising, structural and institutional barriers remain. To bridge the gap between clinical practice and research, it is essential to enhance training opportunities, provide necessary resources, and foster a collaborative environment. Furthermore, aligning recruitment and workforce management strategies with research objectives will be key in creating a cohesive, research-oriented healthcare workforce. By addressing these challenges, IRCCS and similar institutions -recognized globally as research centres and teaching university- can improve workforce stability, enhance quality of care, and ultimately promote scientific progress and innovation.

## Data Availability

The original contributions presented in the study are included in the article/supplementary material, further inquiries can be directed to the corresponding author.

## Footnotes

^1^GRU is a system that integrates a set of tools and procedures used for personnel administration and management within public administrations, including healthcare and hospital institutions in Emilia Romagna region. GRU supports various activities related to personnel management, such as administration of personnel, including contract management, calculation and management of attendance, absences, vacations, and leaves and payroll processing. Additionally, GRU enables resource planning by conducting workforce needs analysis, staff allocation planning, and recruitment management.
